# Influence of Instrument Lubrication on Properties of Dental Composites

**DOI:** 10.1055/s-0042-1743144

**Published:** 2022-04-08

**Authors:** Juliusz Kosewski, Przemysław Kosewski, Agnieszka Mielczarek

**Affiliations:** 1 Department of Conservative Dentistry, Medical University of Warsaw, Warsaw, Poland; 2 Department of Oral Surgery, Medical University of Warsaw, Warsaw, Poland; 3 Department of Conservative Dentistry, Medical University of Warsaw, Warsaw, Poland

**Keywords:** composite modeling, composite resin, dental adhesive, instrument lubricant, modeling resin

## Abstract

Resin composites are one of the most commonly used materials in restorative dentistry. To improve their handling and facilitate restoration sculpting, clinicians began to lubricate modeling instruments with various substances like alcohol, unfilled resins, or even bonding agents. Although the technique is commonly present in daily clinical practice, it has not been precisely described in the literature and both application methods and lubricating materials vary across the available studies. This study aims to summarize the currently available knowledge about influence of instrument lubrication on properties of dental composites. Literature selection was conducted within MEDLINE, SCOPUS, and EBSCO databases. Instrument lubrication seems not to be indifferent for composite mechanical and optical properties. Moreover, various lubricants can differently affect the composite material, so the choice of lubricating agent should be deliberate and cautious. Available *in vitro* studies suggest possible incorporation of lubricant into the composite structure. Unfilled resins based on bisphenol A-glycidyl methacrylate (Bis-GMA) seem to be the best choice for the lubricant, as bonding agents containing hydrophilic molecules and alcohols carry a bigger risk of altering the composite properties. Further research is necessary to evaluate lubricants' influence in clinical practice conditions.

## Introduction

Composite resins are fundaments of contemporary restorative dentistry. 1 Along with evolution of materials and application techniques, composite restorations are capable of closely recreating both the physical and optical properties of natural teeth. As a result, naturally looking, durable, and functional restorations can be provided for both anterior and posterior dentition. 2 3 4

Composite resins present viscous consistency which can impede proper anatomical contour modeling or adaptation of the material to cavity walls. 5 6 To overcome sticking of the composite to hand instruments, lubrication of the instruments with modeling resins, bonding agents, or alcohol became a common practice. Such method deviates from the recommendations of most of the manufacturers. Due to possibility of altering the characteristics of restorative material some authors advise against this technique. 7 Incorporation of other substances into the modeled composite layer may disrupt its composition and influence material properties. 8 9 10

Instrument lubrication technique has not been officially described in the scientific literature, thus lack of standardized research methods to analyze this topic can be observed. Within available *in vitro* studies, authors investigated multiple materials and methods of their application on mechanical and optical properties of restorative materials. 8 11 12 13 14 15 16 17 18 19 20 21 22 23 24 25 26 27

The study aims to summarize the currently available knowledge about influence of instrument lubrication on properties of dental composites. Literature selection was conducted within MEDLINE, SCOPUS, and EBSCO databases. Studies describing usage of resins as a surface glaze after the finished composite polymerization were excluded.

## Resin Lubricants

Widely used group of lubricants meant to facilitate material modeling include resins existing either in clear form, as the so called unfilled resins, or being part of the adhesive systems. They are composed of various methacrylate derivatives present also in organic matrix of dental composites, that is, bisphenol A-glycidyl methacrylate (Bis-GMA), urethane dimethacrylate (UDEMA), triethylene glycol dimethacrylate (TEGDMA), and photoinitiators that allow light curing and in the case of the bonding agent, also additional ingredients facilitating efficient application and adhesion to tooth structure. Hydrophilic molecules present in adhesive systems, such as water, ethanol, or acetone, might affect elements of the composite matrix. 28 29 30

### Physical Properties

Most of contemporarily used composite materials are based on Bis-GMA monomers and comonomers present relatively lower viscosity and molecular weight. 31 32 Organic matrix composition influences handling and physical properties of final product. 5 33 34 Incorporation of additional resin portions with a lubricated instrument raises concerns over possible change in composition of applied composite, leading to a loss of its optimal properties. 8 35 Studies describing influence of instrument lubrication with resins and bonding agents on physical properties of composites are shown in Table 1 .

**Table 1:** Influence of adhesive systems and unfilled resins used as lubricants on the physical properties of composites

Study (year)	Lubricant	Lubricant application method	Composite	Parameters tested	Results
Tjan and Glancy 15 (1988)	Command resin, Bondlite (used with Herculite) HelioBond, Adhesit (used with Heliomolar)	Composite modeling instrument wetted with resin	Herculite Heliomolar	Cohesive strength at the composite interfaces	Lubrication with adhesive decreased Heliomolar strength, no effect on Herculite specimen No effect of lubrication with unfilled resins on cohesive strength of Herculite and Heliomolar
Dunn and Strong 12 (2007)	Unfilled resin (name not mentioned)	Interproximal carver instrument dipped once into a single drop of resin	Filtek Z250	Flexural strength	No influence of lubricant on flexural strength
Barcellos et al 16 (2008)	Composite wetting resin C&B liquid Scotchbond MultiPurpose Adper Single Bond Prime & bond NT	Tip of the instrument painted with the moistured microbrush	Venus	Cohesive strength at the composite interfaces	SBMP lubrication increased cohesive strength, other lubricants had no effect on cohesive strength
Barcellos et al 17 (2011)	Adper SE plus Clearfil SE bond Futurabond M Optibond all-in-one Futurabond NR One up bond F	Tip of the instrument painted with the moistured microbrush	Venus	Cohesive strength at the composite interfaces	Lubrication reduced cohesive strength in all groups except Adper SE Plus
Tuncer et al 20 (2013)	Modeling resin	Round-ended plugger with a diameter of 2-mm dipped into the modeling resin	GrandioSO Gradia direct posterior Aelite LS Posterior Filtek Silorane Aelite all-purpose body Filtek ultimate Clearfil majesty esthetic	Microhardness Surface roughness	Modeling with lubricant and polimerization under polyester strip decreased microhardness and surface roughness Modeling with lubricant with mechanical polishing decreased microhardness and surface roughness of Grandioso Increased surface roughness of Filtek Silorane and Aelite All Purpose
de Paula et al 21 (2016)	ScotchBond MultiPurpose Adper Single Bond	Brush coated with adhesive and the excess removed by stroking onto a glass plate	Filtek Z350 XT Empress Direct	Degree of conversion Cross-linking density	Both adhesives lowered DC and CLD of Empress Direct No significant difference in DC of Filtek Z350 XT SBMP lowered CLD of Filtek Z350 XT
Münchow et al 25 (2016)	ScotchBond MultiPurpose Adper Single Bond	Adhesive applied with disposable microbrush applicator	Filtek Z350 XT	Flexural strength Flexural modulus Microtensile cohesive strength Surface analysis under SEM Water sorption and solubility	ASB reduced flexural strength of composite. Both lubricants: • had no effect on cohesive strength • Reduced loss of cohesive strength, flexural strength and flexural modulus after 6 months • Reduced solubility and water sorption No differences in specimen morphology were observed under the SEM examination
Patel et al 14 (2017)	Optibond FL Optibond solo plus Optibond all-in-one	Plugger dipped into lubricant for 1 second and left to drip/drain for 2 seconds prior to use	Solitaire 2	Diametral tensile strength Water sorption	All tested lubricants increased water sorption and reduced diametral tensile strength of composite
Hamouda 26 (2017)	Tetric N-bond Universal Tetric N-bond	Plugger dipped into lubricant for 1 second and left to drip/drain for 2 seconds prior to use	Tetric N-ceram	Water sorption and solubility	Lubricants increased water sorption of composite No effect on solubility in water
Melo et al 18 (2018)	ScotchBond MultiPurpose Adper Single Bond	Brush coated with adhesive and the excess removed by stroking onto a glass plate	Filtek Z350 XT Empress direct Esthet X HD	Degree of conversion	SBMP reduced DC of all tested composites ASB reduced DC of Empress and increased DC of Esthet X A2
Kutuk et al 19 (2020)	Modeling liquid G-premio bond Optibond XTR primer	Humidified sable brush and microbrush applicator	Essentia Dark Enamel	Microhardness Surface roughness	Optibond XTR lubrication lowered composite microhardness No effect of lubrication on surface roughness
Bayraktar et al 27 (2021)	Modeling liquid Composite primer Modeling resin	Humidified sable brush with excessive material removed using a clean paper tissue	Charisma Smart Estellite Asteria Ceram-X one SphereTEC Admira fusion Filtek ultimate Clearfil majesty ES-2	Microhardness	All of the lubricants reduced composite microhardness

Abbreviations: ASB, Adper Single Bond; CLD, cross-linking density; DC, degree of conversion; SBMP, ScotchBond MultiPurpose; SEM, scanning electron microscope.

#### Composite Resistance

Materials used for replacing dental tissues must be characterized by high and universal resistance to withstand constant occlusal forces. 36 Filler content and monomer types within the composite matrix affect its mechanical properties. 35 37 Changes in precisely selected material composition caused by lubricant incorporation during the application might disturb its internal structure and influence its durability. 3 38

Dunn and Strong have not shown any significant differences in flexural strength between composite samples layered with or without the use of modeling resin. 12 Münchow et al confirmed those observations regarding the use of ScotchBond MultiPurpose (SBMP; 3M ESPE, St Paul, Minnesota, United States) dental adhesive, however, with more hydrophilic bonding agent, Adper Single Bond (ASB; 3M ESPE, St. Paul, Minnesota, United States), flexural strength decreased compared with control group after 24 hours of water storage. Additionally, in presented study, the flexural strength test was repeated after 6 months and the loss of flexural strength was lower than in the control group. Authors explain this by reduction of microporosities on the composite surface; however, scanning electron microscope (SEM) image analyses have not revealed any differences in samples micromorphology. 25

Negative outcome of instrument lubrication on composite resistance was observed by Patel et al. Authors described higher diametral tensile strength (DTS) in control group than in groups where IV-, V-, and VII-generation bonding agents were used as lubricants. 14

#### Composite Tensile Strength

Majority of clinical situations require composite layering because of limited depth of curing light penetration through the material and need of polymerization shrinkage reduction, as well as from esthetic reasons, to compose a restoration from materials with different optical properties. 39 40 Instrument lubrication may lead to changes of material characteristics at the composite layers interface and influence the tensile strength between the increments resulting in lower durability of the restoration. 41 42

Tjan and Glancy observed reduced resistance of Heliomolar (Ivoclar Vivadent, Schaan, Liechtenstein) material lubricated with currently unused bonding system based on polyurethane, Dentine Adhesit (Ivoclar Vivadent, Schaan, Liechtenstein). 15 Polyurethane setting reaction requires the presence of water which can explain obtained low value of tensile strength. 43 Differences in tensile strength have not been noted regarding Heliomolar composite layers applied with the use of Helioseal resin (Ivoclar Vivadent, Schaan, Liechtenstein) which is a combination of Bis-GMA and TEGDMA, as well as within Bis-GMA based Herculite (Kerr, Orange, California, United States). 15

Barcellos et al study has shown reduction of tensile strength between composite layers caused by instrument lubrication with all tested self-etch adhesive systems except for Adper SE Plus Adhesive (3M ESPE, St Paul, Minnesota, United States), adhesive resin from VI-generation system. According to the authors, this is due to fact that ionization of acidic phosphate monomer (MHP) present in Adper SE Plus occurs only in presence of hydrophilic radicals contained in the primer of this system. 17

In earlier studies, the same authors proved that only one out of three tested total-etch adhesive systems, ScotchBond MultiPurpose used during composite modeling caused the increase of cohesive strength of layers in comparison to the control group. ASB, Prime & Bond NT (Dentsply DeTrey, Konstanz, Germany) and modeling resins did not influence this parameter. This relation has been explained by chemical similarity of SBMP and compounds of tested composites, as well as by its lack of hydrophilic solvents. 16

Positive effect of instrument lubrication with SBMP on composite tensile strength was not confirmed during microtensile bond strength (µTBS) test, performed after 24 hours of water storage. Similarly, as in case of flexural strength tests, lubrication with ASB and SBMP decreased tensile strength loss after 6 months of water storage compared with control group. 25

#### Surface Characteristics

Surface quality and smoothness are some of the conditions providing clinical success and durability of created restoration. High gloss of composite surface apart from obvious patient's esthetical satisfaction guarantees also higher staining resistance of the resin. 44 Instrument lubrication during composite application allows to obtain more even surface of freshly placed composite; however superficial modified layer can behave differently during mechanical finishing and polishing. 45

No effect of instrument lubrication with universal bonding agents and modeling resin on nanohybrid composite microhardness was observed by Kutuk et al. Microhardness reduction was only obtained during lubrication with self-etch system primer. Both tested adhesive systems and modeling resin had no effect on surface roughness of the composite. 19

In the study, investigating instrument lubrication with modeling resin, Tuncer et al proved reduction in composite surface microhardness in a group where superficial layer was not mechanically finished. Samples modeled with lubricated instrument but thereafter grinded with aluminum oxide discs have not shown statistically significant difference in surface roughness compared with control for five out of seven examined composites. The results observed were related to removal of resin-rich superficial layer during finishing. Samples modeled with lubricant and polymerized under polyester strip exhibited the lowest surface roughness. Among groups polished after polymerization, instrument lubrication positively affected surface smoothness of Grandioso (VOCO, Cuxhaven, Germany) composite but negatively influenced Filtek Silorane (3M ESPE, St Paul, Minnesota, United States) and Aelite All Purpose Body (Bisco, Schaumburg, Illinois United States) composites. The remaining restorative materials showed no difference compared with the control group. 20

In the research by Bayraktar et al, evaluating the effect of three different modeling resins, composite microhardness reduction was observed for all of the tested composite materials. 27

#### Polymerization Quality

The achievement of desired performance by dental composite materials depends highly on their correct polymerization. 46 Composite's degree of conversion is related to its composition and resin to filler ratio. 47 Usage of higher inorganic filler content and monomers with high molecule weight like Bis-GMA allowed to reduce the polymerization shrinkage of light-cured materials; however, it also affected the reaction kinetics resulting in lower degree of monomer to polymer conversion. 48 49 50 51 Lower degree of conversion has negative outcome on material properties leading to reduced mechanical resistance and decreased color stability. 46 Moreover, high content of unreacted monomer facilitates its release to body environment, limiting material biocompatibility and increasing cytotoxicity. 52 53

In the study conducted by Melo et al, instrument lubrication with SBMP caused reduction in the degree of conversion of all examined composites. Usage of ASB as a lubricant also decreased the degree of conversion for Empress Direct (Ivoclar Vivadent, Schaan, Liechtenstein) A2 and Bleach shades however increased it for Esthet X HD (Dentsply Caulk, Milford, Delaware, United States) A2 shade. 18 De Paula et al during analysis of the same adhesives have not observed significant effect of instrument lubrication on composites degree of conversion; however, lower cross-linking density has been noted with SBMP as a lubricant. 21

The amount of available studies verifying effect of instrument lubrication on polymerization quality is limited. Results suggest possible incorporation of lubricant particles into composite matrix and alterations in the polymerization reaction kinetics.

#### Scanning Electron Microscope Analysis

During cross-section surface analysis of freshly placed composite samples using SEM, no changes in the micromorphology of interlayer interface have been observed. 23 25 Analysis performed after 12 months of wine storage revealed heterogenic structure with evident connection layer in groups where instruments were lubricated with adhesives. 23

Superficial composite layer examination after wine storage showed lower surface degradation in the group modeled with adhesive system as a lubricant compared with the control group. Authors connect that surface degradation with influence of the alcohol contained in wine. 23 This hypothesis can be confirmed by lack of visible surface degradation of composite modeled with lubricated instrument but stored in coffee solution observed in another study. 19

#### Water Sorption and Solubility

Composite restorations in the in vivo conditions are permanently interacting with surrounding water solution: composite absorbs little amounts of water and also its small particles get dissolved during the hydrolytic reaction. 54 55 Increased water sorption and solubility negatively affect mechanical properties of composite material. 56 Those parameters rely mainly on composition and structure of composite organic matrix phase, 57 therefore incorporation of additional monomers and hydrophilic substances during modeling with lubricated instrument creates a possibility of significant changes in terms of composite stability in mouth environment. 58

The use of SBMP and ASB as instrument lubricants reduced composite solubility compared with control group. Water sorption was reduced only with the use of SBMP. 25 In another study, no effect of modeling with V- and VI-generation adhesive systems on composite solubility was also observed. 26

Different outcome was obtained during measurements of water sorption, it has increased in all tested groups. Additionally, the study has shown no linear relation between number of layers modeled with lubricant and water sorption. 26 Higher water sorption was also confirmed for instrument lubrication with V- and VII-generation bonding agents from another manufacturer and with adhesive resin from VI-generation system. 14

### Optical Properties

Composite modeling with lubricated instruments or brushes is especially helpful during work in the esthetic region. Replication of correct anatomical form and proper surface texture already at the application stage allows clinician to save time needed for contouring and finishing of the restoration. Qualitative and quantitative compositions of organic matrix affect color, translucency, light refraction index of dental resins, and also their staining susceptibility. 59 60 61 Changes within those parameters can determine failure in esthetic integration despite the right restoration shape. 62

Studies examining influence of instrument lubrication with modeling resins and adhesive systems on composite optical properties are listed in Table 2 .

**Table 2:** Influence of adhesive systems and unfilled resins used as lubricants on the optical properties of composite

First author	Lubricant	Lubricant application method	Composite	Parameters tested	Results
Tuncer et al 20 (2013)	Modeling resin	Round-ended plugger with a diameter of 2-mm dipped into the modeling resin	GrandioSO Gradia direct posterior Aelite LS posterior Filtek Silorane Aelite all-purpose body Filtek Ultimate Clearfil majesty esthetic	Color stability after thermocycling	Lubrication Increased color change of: • Polished Filtek Silorane • Filtek Ultimate and Filtek Silorane polymerized under polyester strip • Lubrication reduced color change of: - Polished Aelite all-purpose body and Aelite LS posterior • Clearfill Majesty, Aelite All Purpose Body, Aelite LS Posterior polymerized under polyester strip
Münchow et al 25 (2016)	ScotchBond MultiPurpose Adper Single Bond	Adhesive applied with disposable microbrush applicator	Filtek Z350 XT	Color stability Transparency parameter	SBMP lubrication caused increased color change after 180 days of water storage but lowered color change after 180 days of wine storage ASB lubrication increased color change after 1 day of water storage Lubrication with both adhesives increased translucency
Sedrez-Porto et al 23 (2016)	ScotchBond MultiPurpose	Instrument not mentioned	Filtek Z350 XT	Color stability	SBMP lubrication reduced color change after 6 and 12 months of wine storage for both polished and unpolished specimen
Sedrez-Porto et al 24 (2017)	ScotchBond MultiPurpose Adper Single Bond	Adhesive applied with disposable microbrush applicator	Filtek Z350 XT	Color stability Transparency parameter	SBMP lubrication reduced color change after 6 and 12 months of wine storage Lubrication with both adhesives had no influence on translucency after 6 and 12 months of water storage
Araujo et al 22 (2018)	ScotchBond MultiPurpose Adper Universal	Brush covered with the adhesive	Filtek Z250	Color stability Transparency parameter	Lubrication with Adper universal reduced color change during storage in staining solutions Lubrication with both adhesives had no influence on translucency
Melo et al 18 (2018)	ScotchBond MultiPurpose Adper Single Bond	Brush coated with adhesive and the excess removed by stroking onto a glass plate	Filtek Z350 XT Empress direct Esthet X HD (A2 and bleach shades)	Color stability Transparency parameter	SBMP lubrication increased color change of Esthet X HD Lubrication with both adhesives increased translucency of Filtek Z350 XT A2 shade Lubrication with ASB decreased translucency of Esthet X HD bleach shade

Abbreviations: ASB, Adper Single Bond; SBMP, ScotchBond MultiPurpose.

#### Translucency Change

Increased translucency of Filtek XT Z350 (3M ESPE, St Paul, Minnesota, United States) and Filtek Z250 (3M ESPE, St Paul, Minnesota, United States) was described in the literature while using the instrument lubrication technique with bonding agents from the same manufacturer, ScotchBond MultiPurpose and ASB. 18 22 24 25 In the research conducted by Melo et al, composites from other producers were also included, Empress Direct and Esthet X HD. Their translucency got decreased or was not affected by modeling with lubricated instrument. 18

#### Color Change

Color stability is one of the requirements for restorative materials. Pigments contained in daily diet can cause external and internal discoloration both within the dental tissue and in the composite. 63

Tuncer et al examined effect of instrument lubrication with Modeling Resin (Bisco, Schaumburg, Illinois, United States) on color change of composites. Higher color change range was observed after thermocycling Filtek Ultimate (3M ESPE, St Paul, Minnesota, United States) and Filtek Silorane samples; however, only for the latter change was greater than estimated acceptation threshold. 64 Clearfill Majesty (Kuraray Medical Inc., Tokyo, Japan), Aelite LS posterior (Bisco, Schaumburg, Illinois United States), and Aelite All-Purpose Body composites have shown lower color change compared with control group. Instrument lubrication with SBMP and ASB adhesives caused higher color change after water storage. 24 25 Melo et al observed increased color change only for one out of three tested composites. 18

Instrument lubrication with SBMP, ASB and Adper Universal (3M ESPE, St Paul, Minnesota, United States) resulted in lower composite color change after storage in strongly staining solutions. 22 23 25

## Alcohol Lubricants

Usage of alcohol as a substance preventing sticking of the chemically cured composite to plastic modeling instruments was for the first time described already over 40 years ago. Instruments moistened in alcohol did not affect material adaptation to cavity walls and composite resistance. 65 However, it has also been proven that too large contamination of composite resin with alcohol drastically worsens its mechanical properties. 66 Research results regarding instrument lubrication with alcohol are listed in Table 3 .

**Table 3:** Influence of alcohol used as a lubricant on composite properties

First author	Lubricant	Lubricant application method	Composite	Parameters tested	Results
Tjan and Glancy 15 (1988)	70% ethanol 70% isopropanol	Modeling instrument moistured with alcohol	Herculite Heliomolar	Cohesive strength at the composite interfaces	Lubrication with alcohols decreased Heliomolar cohesive strength, no effect on Herculite specimen
Patel et al 14 (2017)	Ethanol	Ball burnisher dipped in alcohol for 1 second and left to drip for 2 seconds	Solitaire 2	Diametral tensile strength Water sorption	Lubricant decreased diametral tensile strength and increased water sorption
Dunn and Strong 12 (2007)	70% isopropanol	Interproximal carver instrument wiped with an alcohol saturated gauze	Filtek Z250	Flexural strength	Lubricant had no influence on flexural strength
De Paula et al 21 (2016)	70% ethanol 100% ethanol	Spatula left for 3 seconds on a gauze with dispensed alcohol	Filtek z350 XT Empress Direct	Degree of conversion (DC) Cross-linking density (CLD)	Reduced DC of Empress Direct in 70% ethanol group No effect of 100% ethanol on DC No effect of 70% and 100% ethanol on CLD

Tjan and Glancy have shown negative influence of instrument lubrication with alcohol during layering of UDEMA-based composite on tensile strength. Such relation was not observed for composite based on Bis-GMA, where samples during the tests sustained mostly cohesive fractures. Different behavior of those two materials can be explained by different vulnerability to degradation by hydroxyl ions contained in alcohol. 28 During fracture site analysis, the presence of white spots on layer's connection surface was observed in samples treated with ethanol. Authors suggest that this can be an effect of precipitation of filler particles after dissolution of resin matrix. 15 This phenomenon was also observed later in another study. 14

Negative influence of instrument lubrication with alcohols on physical properties of layered composite was described by other researchers. Patel et al demonstrated composite resistance decrease in diametral tensile strength test for samples applicable with the use of 70% ethanol and also their increased water sorption compared with control group. 14 Different outcome was shown by Dunn et al, suggesting no effect of instrument lubrication with the same ethanol concentration on resin's flexural strength. 12 Conflicting results can be explained by differences in lubricant application methods. In experiment by Patel et al, the instrument was dipped in alcohol solution while in the study by Dunn et al, the instrument was only wiped with alcohol moistened gauze. Rapid ethanol evaporation from the instrument surface in room temperature could be the reason why it had no effect on composite material during modeling. Similar doubts about presence of lubricant on the instrument using absolute ethanol were described by de Paula et al. The authors observed reduction in degree of conversion of the Empress Direct composite while using 70% ethanol but no effect on that parameter with use of absolute ethanol. Additionally, there was no effect of instrument lubrication with 70 and 100% ethanol on conversion degree of Filtek XT 350 composite and on the polymer cross-linking density of both tested composites. 21

Research results differ according to the application method used and composite type. The amount of alcohol present on the instrument during the modeling can be significantly different in mentioned studies. Furthermore negative effect of instrument lubrication with alcohol seems connected with its quantity introduced into composite portion, what is consistent with the observations of Sneed and Draughn. 66

## Discussion

The quoted results present current knowledge about influence of instrument lubrication with resins, bonding agents, and alcohol on properties of dental composites.

From all tested lubricants the least negative effects on mechanical properties of composites could be observed for resin with composition similar to composite organic matrix, as well as in case of alcohols, presence of hydrophilic particles in adhesive systems can more affect the composite properties compared with bonding agents with more hydrophobic composition and unfilled resins. 29 Negative influence of instrument lubrication with methacrylate-based resins on mechanical behavior of Filtek Silorane composite which is based on silorane matrix can additionally suggest the importance of chemical compatibility between used lubricant and composite material.

Changes in conversion degrees and microhardness values of superficial composite layers indicate incorporation of lubricant particles into the composite structure and consequently creation of external resin-rich layer. 49 That layer is probably thin enough to be completely eliminated during standard finishing and polishing, which are inherent steps of each restorative procedure. As a result, the possible influence of that layer on finished composite surface properties can be most likely omitted. 20

Most often examined resin was ScotchBond MultiPurpose adhesive, consisting mostly of Bis-GMA and hydroxyethyl methacrylate (HEMA). Viscosity of monomer mixture has a direct effect on degree of conversion and polymerization shrinkage of composites. 47 Modifications in Bis-GMA percentage influence resin polymerization parameters. 47 67 68 HEMA as a light molecular weight and low viscosity monomer promotes increase in conversion degree due to higher particle mobility during polymerization process. 69 At the same time, HEMA is binding only in linear positions, not creating cross-chains which results in higher susceptibility to hydrolysis. 54

Instrument lubrication does not seem to have a noticeable effect on base color of methacrylate composites when both the material and the lubricant present similar monomer composition. The increase in staining resistance of composites after modeling with resin lubricated instrument is an interesting relationship. This positive outcome might be mainly connected to improved composite adaptation and reduction of surface microdefects. As a result, a material sealing effect is obtained, similarly to surface glaze application on already cured composite. 70 It is worth mentioning that studies regarding optical properties did not include parameters, such as light refraction index or fluorescence, the properties of high clinical significance that allow to achieve natural esthetics and metamerism of composite restorations. 4 71

The use of ethanol and isopropanol carries high risk of damaging composite resin matrix elements which can cause decrease in tensile strength between the layers and surface degradation. Observed lack of effect of ethanol on composite properties might be a consequence of a full evaporation of that substance from the modeling instrument. As a result, it cannot be equated to instrument lubrication but rather just instrument cleaning and degreasing.

## Conclusion

The topic of instrument lubrication during composite sculpting has been discussed from several dozen years. Usage of that technique in everyday clinical practice is a fact, despite the doubts around its safety and influence on applied material. Methodological differences present in particular studies involved in this analysis increase the number of variable parameters, in example type of the instrument used to smoothen the composite surface which additionally impede direct results comparison. According to current research, the following inferences can be drawn:

- The composition of the lubricant can influence properties of applied composite.
- Usage of alcohol as an instrument lubricant carries a risk of damaging the resin matrix and consequently decreases the mechanical performance of the material.
- Among analyzed bonding agents, the least negative effects were observed after use of adhesive resins, which are free from hydrophilic particles, results were close to those obtained with the use of dedicated modeling resins.
- Methacrylate based lubricants do not seem to negatively influence the optical properties of the most of composite materials; however, they are not recommended for work with silorane matrix based composites.
- Instrument lubrication technique allows to obtain more regular surface, with less microporosities and imperfections what positively affects composite staining resistance and stability in water environment.

An important issue that has not been yet answered is the real amount of lubricant incorporated into the composite structure and consequent potential modification of its chemical composition taking into consideration an accurate representation of clinical conditions.
